# Consumption of the Soluble Dietary Fibre Complex PolyGlycopleX^®^ Reduces Glycaemia and Increases Satiety of a Standard Meal Postprandially

**DOI:** 10.3390/nu8050268

**Published:** 2016-05-06

**Authors:** Vicky A. Solah, Babette O’Mara-Wallace, Xingqiong Meng, Roland J. Gahler, Deborah A. Kerr, Anthony P. James, Haelee K. Fenton, Stuart K. Johnson, Simon Wood

**Affiliations:** 1 School of Public Health, Faculty of Health Sciences, Curtin University, Perth, Western Australia 6845, Australia; babette.omara-wallace@postgrad.curtin.edu.au (B.O.-W.); d.kerr@curtin.edu.au (D.A.K.); T.P.James@curtin.edu.au (A.P.J.); h.fenton@curtin.edu.au (H.K.F.); s.johnson@curtin.edu.au (S.K.J.); simonwood@shaw.ca (S.W.); 2 Flinders Centre for Innovation in Cancer, School of Medicine, Flinders University, Adelaide 5001, Australia; rosie.meng@flinders.edu.au; 3 Factors Group R & D, Burnaby, BC V3N 4S9, Canada; rgahler@naturalfactors.com; 4 InovoBiologic Inc., Calgary, AB Y2N 4Y7, Canada; 5 Food, Nutrition and Health Program, University of British Columbia, Vancouver, BC V6T 1Z4, Canada

**Keywords:** PGX, dextrin, glycaemia, fibre, satiety

## Abstract

The effect of consumption of PolyGlycopleX^®^ (PGX^®^) was compared to wheat dextrin (WD) in combination with a standard meal, on postprandial satiety and glycaemia in a double-blind, randomised crossover trial, of 14 healthy subjects trained as a satiety panel. At each of six two-hour satiety sessions, subjects consumed one of three different test meals on two separate occasions. The test meals were: a standard meal plus 5 g PGX; a standard meal plus 4.5 g of PGX as softgels; and a standard meal plus 5 g of WD. Subjects recorded fullness using a labelled magnitude scale at 0, 15, 30, 45, 60, 90, and 120 min and the total area under the curve (AUC), mean fullness *vs.* time was calculated. The meals with PGX (in granular and softgel form) gave higher satiety (AUC) (477 ± 121 and 454 ± 242 cm·min), than the meal with WD (215 ± 261 cm·min) (*p* < 0.001). Subjects had blood glucose levels measured after the meals with PGX (granules) and WD. Glucose response (AUC) was significantly lower (*p* < 0.001) after the PGX meal than for the WD meal.  The high viscosity reported for PGX is a likely mechanism behind the significant satiety and blood glucose modulating effects observed in this study.

## 1. Introduction

### 1.1. Health Benefits of Dietary Fibre

The health benefits of dietary fibre have been observed for centuries, with Hippocrates recognising its role in improving bowel function [1]. Both observational and intervention-based studies have shown that dietary fibre is protective against a range of disorders including obesity, type 2 diabetes mellitus, and colon cancer [2]. A key rheological feature of many soluble fibres is viscosity and it has been hypothesised that viscous fibres exert their physiological health benefits by increasing the viscosity of the gastrointestinal contents; thus inhibiting nutrient-enzyme interactions [3,4]. Soluble fibres such as pectin, gums, and low molecular weight non-starch polysaccharides have a recognised role in reducing digestion rates, increasing satiety, lowering cholesterol and moderating postprandial blood glucose levels [5]. Soluble fibres such as guar gum and wheat dextrin (WD) (a digestion resistant glucose polymer) are generally fermented in the large intestine where they have a beneficial effect on the composition of the intestinal flora and help normalise bowel function [6,7]. By prolonging the transit time through the gastrointestinal tract and reducing the rate at which nutrients are digested and absorbed from the small intestine, viscous fibres are able to increase satiety and attenuate postprandial glucose responses [3,8].

### 1.2. Satiety and Soluble Fibre

Satiety has been described as the feeling of being satisfied after an eating episode, resulting in delayed onset of the next meal [9], and thus potentially helping to modulate weight gain. In a study investigating the effect of soluble fibre viscosity on hunger and satiety, Slavin and Green [10] found that regardless of dose, soluble fibres that were not viscous had no effect on these parameters. Similarly, randomised-control trials by Mattes and Rothacker [11], Marciani *et al.* [12], and Hoad *et al.* [13], found that when solutions of different viscosities were administered, those with higher viscosities prolonged satiety and reduced hunger most effectively. Likewise, a review of 15 trials testing viscous fibre from psyllium, oatmeal, legumes, and guar gum, determined all fibres tested were able to increase satiety and reduce subsequent food intake [14]. Viscous fibre has additionally been found to be more effective at increasing satiety when volumes of water greater than 200 mL have been provided as part of the dose. Lafond *et al.* [15] found no differences in postprandial appetite response for a ready-to-eat cereal with added arabinoxylan of different viscosities and consumed with added liquid, but suggested that the fibre may have needed to be more fully hydrated for viscosity to have affected the satiety response. In earlier studies, a high viscosity alginate-based powder in 250 mL water reduced hunger and provided a higher satiety effect than a lower viscosity 250 mL protein drink [16]. Guérin-Deremaux *et al.* [17] reported that NUTRIOSE, a low viscosity soluble dextrin, produced improved short-term satiety and hunger. This effect of dextrin on satiety indicates that mechanisms other than viscosity, such as changes to blood glucose levels, may contribute to the satiety effects of dietary fibres. 

### 1.3. Glycaemia and Soluble Fibre

Soluble viscous fibres have a positive effect of reducing postprandial glycaemia and thus may have a role in managing and preventing type 2 diabetes [18,19]. The correlation between fibre viscosity and reduced postprandial blood glucose levels is well established and early studies proposed that this beneficial effect was due to the delayed rate at which glucose was absorbed from the small intestine [3]. This hypothesis has since been extended to consider the not only glycaemic index (GI) of ingested foods, but increased viscosity and the improved hormonal response to nutrients, as other potential mechanisms by which viscous fibres exert their effect [20,21,22]. When combatting major chronic disorders, such as obesity and type 2 diabetes, evidence suggests that fibre-enriched products have an important role, but fibre intakes have remained at approximately half the recommended level for the past decade [23]. Fibre supplementation and increased consumption of everyday high fibre food products containing functional fibres may play an important role in satiety, reducing the drive to eat, and potentially assisting weight loss [24]. Many different types of fibres have been reported as having satiety and glycaemia benefits but research on the mechanisms involved in these responses and the degree of their benefit to health is limited.

### 1.4. PGX and Wheat Dextrin

PolyGlycopleX^®^ (PGX) is a soluble viscous non-starch polysaccharide-based complex manufactured from konjac glucomannan, sodium alginate, and xanthan gum using a proprietary process (EnviroSimplex^®^) [20]. PGX when mixed with water results in higher viscosity than other single dietary fibre sources when mixed with water [20]. PGX can be consumed as granules or in a softgel form, which are gelatin-based capsules containing PGX and medium chain triglycerides as a paste. There is evidence that PGX reduces glycaemia, with studies by Brand-Miller *et al*. [21,22] demonstrating that the addition of PGX reduced GI when added to foods and the 120 min area under the plasma glucose curve was reduced by 50%.

The varying degrees of effect reported on different fibres for satiety and food intake may reflect their differences in solubility and viscosity. Smith *et al.* [20] compared the viscosity of different fibre types, such as guar and psyllium, to PGX (0.5% w/w) and noted that PGX had the highest viscosity (>80 cP) after hydration followed by stirring at 658 rpm over a period of 25 min. PGX consumed as a meal replacement drink in adolescents was reported to reduce hunger levels and *ad libitum* food intake more effectively when compared to a consumption of a lower viscosity cellulose beverage [25]; and PGX resulted in greater satiety than low viscosity inulin when both were consumed with 500 mL water [26]. In addition to viscosity, a 2014 study demonstrated a dose effect, where 2.5 g of PGX showed a strong effect on satiety and a 7.5 g dose showed a greater effect [26]. 

However, it is not only the viscosity of the dietary fibre source that has demonstrated glycaemia modulating effects. Slavin *et al*. [7] reported that WD, a soluble, starch based, indigestible polysaccharide which is formed when starch is heated and treated with enzymes, was effective in stabilising blood glucose [7]. WD has been shown to modulate the glycemic response, as well as improve short-term satiety [27,28,29].

The objective of this study was to assess the use of a trained satiety panel to compare the effects of granular PGX with WD on postprandial satiety and glycaemia, and compare the effects of granular and softgel PGX on postprandial satiety. Research has previously demonstrated the benefits of using a trained panel for satiety evaluation, in terms of increasing the precision of the evaluation [30] and thus allowing smaller but clinically relevant differences between treatments to be observed. The effect of WD versus PGX is of merit since the two fibre sources have both demonstrated potentially beneficial effects on satiety and glycaemia but their mechanism of action is likely to be different due to their differences in viscosity.

## 2. Experimental Section

### 2.1. Subjects

Fourteen healthy adults (2 male and 12 female); age 21.9 ± 4.0 years (range 19–32 years); and body mass index 23.2 ± 4.3 kg/m^2^ (range 19–32 kg/m^2^), participated in this study at Curtin University, Perth, Western Australia. Flyers, posters, information sessions, internet, and radio announcements were used to recruit subjects. Subjects were excluded if they were: smokers; pregnant; had food allergies or a history of cardiovascular disease; consumed excessive amounts of alcohol; or were taking medications or dietary supplements known to affect satiety. Screening questionnaires were used to assess these criteria and acceptance letters were sent to eligible candidates. Subjects were also screened to ensure a low level of dietary restraint, disinhibition, and perceived hunger using a Three-Factor Eating Questionnaire [31]. The trial was registered with the Australian New Zealand Clinical Trials Registry (ACTRN12614000911695). The Human Research Ethics Committee of Curtin University approved the study (HR03 2014) and informed written consent was obtained from all subjects prior to commencement of the trial. 

### 2.2. Study Design

**Figure 1:**
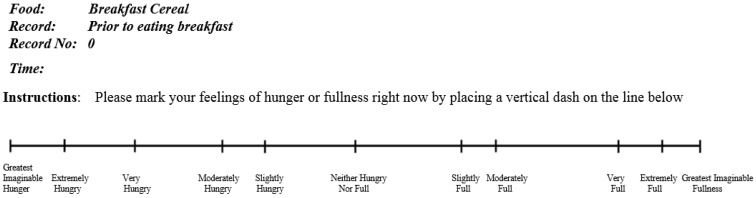
Labelled magnitude scale used to assess satiety.

The study was a randomised, crossover trial with the PGX and WD arm double-blinded. The third arm, PGX softgel was randomised, single-blinded. Subjects participated in six breakfast and two-hour postprandial sessions, held fortnightly. At the initial visit, subjects were assigned a three-digit number and randomly allocated a unique meal sequence. The 19.0 cm labelled magnitude scale (LMS) [30] (Figure 1) was used as the satiety measurement tool. At the initial visit, subjects were trained in the use of the LMS by a primary researcher using a pre-defined scripted protocol and consensus was reached by the subjects (satiety panel) on the meaning of the LMS descriptor words [30]. The LMS was considered to provide better discrimination of satiety sensations compared to a visual analogue scale (VAS) for the trained panel, although it is important to consider “hunger” separately to “fullness” [32]. At each session, subjects consumed one of three different test meals on two occasions: a standard meal (1205 kJ) plus 5 g PGX; the standard meal plus 4.5 g of PGX as six softgels (each containing 750 mg PGX and 600 mg medium chain triglycerides); and the standard meal (1205 kJ) plus 5 g WD (Benefibre, Novartis Consumer Health Australasia, Pty, Ltd., Mulgrave, Victoria, Australia) (Table 1). The standard meal was Special K Original and Cornflakes (Kellogg’s, Ferntree Gulley, Victoria, Australia) mixed together in equal portions (total, 45 g) and full cream milk (175 mL). 

On each test morning, subjects arrived at the School of Public Health, Curtin University having fasted for 10 h overnight and consuming only *ad libitum* water. Subjects were then given instructions about the testing protocol and had their finger prick fasting blood glucose measured by a glucometer in the clinic room. Subjects were then seated in individual sensory booths in the sensory evaluation laboratory where the breakfast test meals were served. Subjects rated their feeling of hunger and fullness at baseline (before eating commenced) using the LMS. The subjects were then provided with the test meal which consisted of the standard breakfast meal (Table 1), a 500 mL bottle of water, and a small plastic cup containing either 5 g of WD, 5 g of PGX, or 4.5 g of PGX in the form of six softgels. Subjects sprinkled the fibre onto the meal prior to eating or swallowed the softgels and consumed the entire test meals including 500 mL of water within 12 min. 

After consumption of the test meal, subjects moved to an adjacent room (where they were not allowed to eat or drink but were allowed to use a computer or read) to rate their sensation of fullness using the LMS at 15, 30, 45, 60, 90, and 120 min after commencement of eating. Immediately after each satiety time point, subjects moved to the clinic room where finger prick blood samples were taken and tested for blood glucose levels (BGL). 

### 2.3. Satiety Measurement

A subjective hunger or fullness score was assessed using the validated labelled magnitude scale (LMS) [30]. During testing, the panel was reminded that hunger involves the desire to eat and fullness involves feelings of a physical stretch in the stomach [30,33]. Vertical lines on the scale had labelled anchors that ranged from “Greatest Imaginable Hunger” to “Greatest Imaginable Fullness” (Figure 1). Panelists marked anywhere along the scale to match their perceived intensity of hunger or fullness; and a separate LMS was used for each time point. The LMS marks were enumerated by measuring from the centre point of the scale, which is represented numerically as 0, to where the panelist marked. The “Greatest Imaginable Hunger” therefore equated to −9.5 cm and “Greatest Imaginable Fullness” to +9.5 cm. 

### 2.4. Capillary Blood Glucose Measurement

Finger prick blood samples (several drops) were collected at each postprandial time point using a HemoCue Glucose RT microcuvette (HemoCue Australia Pty Ltd., Wamberal, Australia). The glucose concentration of the capillary blood samples were analysed using a Glucose 201 RT analyser (HemoCue Australia Pty Ltd., Tumbi Umbi, NSW, Australia) which had been calibrated with a Glucotrol solution (HemoCue Australia Pty Ltd., Tumbi Umbi, NSW, Australia).

### 2.5. Viscosity of PGX

The viscosity (cP) of PGX and WD was measured using a Brookfield (RVT) viscometer with spindle number 3 (Brookfield Engineering Labs Inc., Stoughton, MA, USA). PGX granules (5 g) or the PGX paste (5 g removed from softgel) was blended at 25 °C with 350 g deionised water for 30 s at 4000 rpm and then for 30 s at 8000 rpm. The settings used for PGX from the softgel, were 10 rpm from 0 to 5 min and then 1 rpm was used until 120 min to obtain readings. The settings used for PGX granules were 50 rpm from 0 to 10 min, then 10 rpm until 30 min followed by 1 rpm until 120 min. The viscosity of WD, 5 g in 350 g deionised water was measured using spindle 1 and 3 at 1000 rpm for 30 min.

### 2.6. Statistical Analysis

Data were generated from duplicate testing of three meals. The normality of outcome variables was tested and confirmed as a normal distribution. The total area under the curve (AUC) of the postprandial satiety response of the fullness rating (cm) *vs.* time (min), and blood glucose levels (mmol/L) *vs.* time, was calculated using the trapezoidal rule [26]. The overall treatment effect of PGX was assessed by comparing AUC for satiety and glucose using a mixed effect model. The total AUC of satiety or blood glucose level was the dependent variable, while accounting for the correlation between assessments at six test occasions made by the same subject. A *p* value < 0.05 was regarded as statistically significant. The results from the mixed model were presented as the mean differences between trial groups, SD with 95% confidence interval, and *p* values. All analyses were undertaken using Stata statistical software (MP 13.1, StataCorp, College Station, TX, USA).

## 3. Results

### 3.1. Satiety

**Figure 2:**
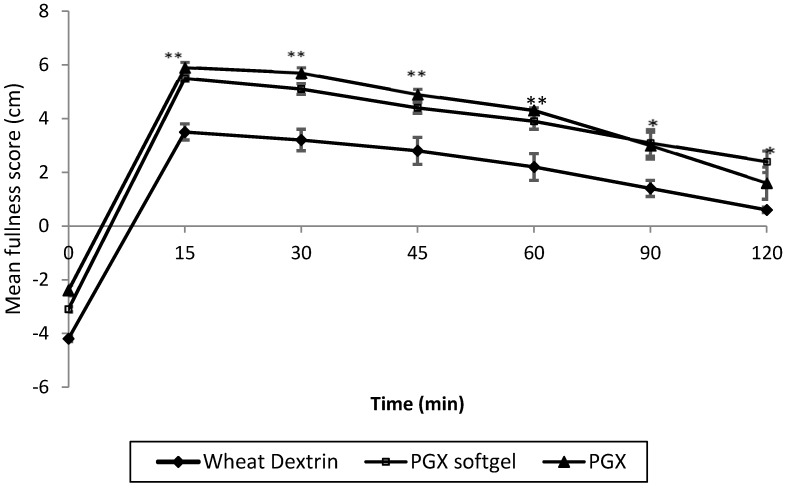
Comparison of self-reported fullness scores (mean ± standard error of mean, SEM) from labelled magnitude scale (LMS) for WD with PGX softgel and PGX (granules). ** fullness score for PGX and PGX softgel were significantly different (*p* < 0.01) to WD. * Only PGX softgel was significantly different to WD (*p* < 0.01).

The self-reported fullness scores from the labelled magnitude scale (LMS) over time by treatment group for the PGX, PGX softgel, and WD meals are presented in Figure 2. The mean fullness score peaked at 15 min and then dropped over the time period of 30 to 120 min for all groups. The mean fullness score of the PGX softgel meal for all time points was significantly higher than that of the WD meal (Figure 2, all *p* < 0.01). The mean fullness score for the PGX (granules) meal was also significantly higher at time points 15, 30, 45, and 60 min (5.9, 5.7, 4.9, 4.3 respectively) than for the WD meal (3.5, 2.8, 2.2, 1.4 respectively) (all *p* < 0.01) and showed a strong trend towards being higher at 90 min (*p* = 0.051). Mean fullness scores at each time point from the PGX group did not differ significantly from those of the PGX softgel group. The AUC for postprandial satiety (subjective hunger/fullness score *vs.* time) for PGX meal and PGX softgel meal were significantly higher than for the WD meal (Table 2, both *p* < 0.001). There was no significant difference in the AUC of satiety between the PGX and the PGX softgel meal groups (*p* = 0.81). 

### 3.2. Blood Glucose

**Figure 3:**
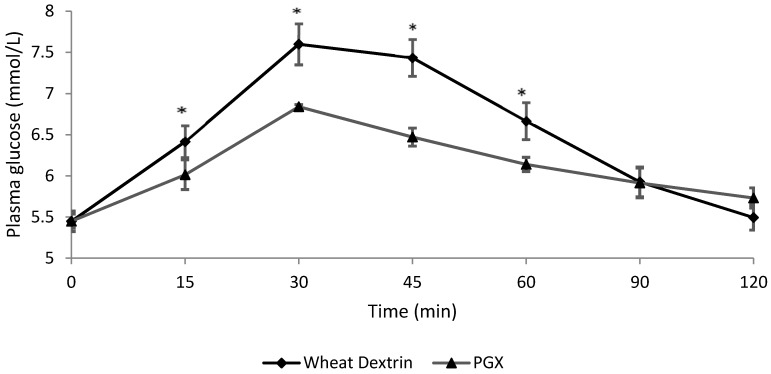
Comparison of postprandial blood glucose response (mean ± SEM) over time by treatment group for wheat dextrin (control) and PGX (granules). * PGX plasma glucose level was significantly different to that of WD (*p* < 0.05).

The postprandial capillary blood glucose responses of the PGX (granule) meal and WD meal are presented in Figure 3. Only the granular form of PGX was used for postprandial blood glucose response measurement. The time to peak blood glucose level for both PGX and WD was 30 min. At 15, 30, 45, and 60 min after meal consumption, the blood glucose mean values for the PGX meal were significantly lower (6.00, 6.84, 6.47, 6.14 respectively) than those for the WD meal (6.42, 7.60, 7.43, 6.67 respectively) (*p* < 0.05). At 120 min, the effect reversed and the mean blood glucose level was slightly but significantly higher for the PGX than for the WD meal (*p* = 0.008). Table 2 reports the mean AUC for postprandial blood glucose levels which was significantly lower for the PGX meal than for the WD meal. 

### 3.3. Viscosity

The PGX softgel demonstrated higher viscosity than that of the PGX (granules) after 10 min of mixing (35,460 cP and 1050 cP respectively) and 120 min (90,000 cP and 54,850 cP respectively). The viscosity of the WD was below the limit of detection of the viscometer, demonstrating its very low viscosity.

## 4. Discussion

### 4.1. Postprandial Satiety

Results from this study showed that when PGX in the form of granules or softgel was co-consumed with a standard meal, the postprandial satiety (AUC) was higher, compared to WD co-consumed with the same meal. Several factors may have contributed to the higher satiety response of the PGX meals; these include the mixing and hydration of PGX with fluids in the stomach, the rate of dissolution of PGX, and the final viscosity of PGX before and after stomach emptying.

Understanding the mechanism of how a fibre, such as PGX, affects satiety is complex, particularly in mixed meal studies [34] as in the present research. The mechanism and timeframe for dissolution of water-soluble polysaccharides, and the rate and magnitude of viscosity developed, are key factors influencing the effectiveness of soluble fibre on increasing satiety [20]. Different soluble viscous fibres have different hydration rates which impact the ability and time that it takes for maximum viscosity to be reached, particularly when they are consumed in the unhydrated form. Some guar gums take up to five hours to reach 60% of their maximum viscosity, which is beyond the time relevant to stomach emptying of 4.5 h [20,35]. Subjects in this research consumed 500 mL water with the PGX meals and the viscosity results presented suggests that the meals containing PGX could become viscous in the stomach. In the meal containing the non-viscous WD, any resulting viscosity in the stomach would only be provided by the breakfast cereal in the standard meal. Research investigating the development of viscosity from polysaccharides [20] showed PGX forms a viscous matrix in sufficient time to influence feelings of fullness. In a study examining the effect of konjac glucomannan addition, on *in vitro* dairy protein digestion, it was reported that the konjac glucomannan addition led to high apparent viscosity aggregates [36] suggesting that the konjac glucomannan in PGX could also contribute to higher viscosity aggregates during digestion. Furthermore, in the same study of adding hydrocolloids to dairy proteins *in vitro* [36], the addition of alginate was hypothesised to have slowed down the rate of protein digestion and therefore delayed gastric emptying; both of these findings support the postprandial satiety effect of PGX.

Peptide YY (PYY) is a physiological gut-derived satiety hormone, which slows gastric emptying, regulates appetite, and is released in response to a meal [37]. PYY has been shown to increase in the blood in the first 15 min following PGX consumption and peaks at 1–2 h [38]. The effects of increased PYY may therefore have contributed to the satiety effect of PGX found in the present study. Another mechanism for elevated PYY levels after PGX consumption may be via PGX microbial fermentation, as products of fermentation may stimulate the release of PYY [30,39].

When considering the satiety effects of the different forms of PGX, granules versus softgel, the nature of the food with which they are consumed must also be considered. The mouthfeel, flavour, and odour of the food may influence the pleasantness and palatability of the two forms of PGX and this in turn may have influenced the satiety responses [40]. In the present study, the satiety effect of PGX did not differ from that of PGX softgel, however since the PGX in the softgels demonstrated higher viscosity, this form of PGX may have greater potential to provide increased satiety under different meal conditions. It was observed by the researchers that there was little change to the structure of the PGX granules during the meal consumption time (<12 min) indicting a lack of full hydration. This was possibly due to the fibre being dispersed in the breakfast cereal flakes and the milk being cold (5 °C) limiting fibre hydration and swelling. Alternately, the breakfast cereal may have been hydrating more rapidly and thus competed for the water in the meal. Subjects in the present research consumed 500 mL of water with the standard meal, which assisted in the hydration of the PGX and formation of a viscous matrix in sufficient time to influence feelings of fullness. The time taken for the PGX to fully hydrate and reach maximum viscosity, however appears to be longer than 120 min and therefore it is suggested that full hydration of the PGX may produce different effects from the two PGX forms, granules and softgels, on satiety. PGX softgels have been found to be effective at providing extended satiety effects in previous research where subsequent food intake was measured [32]. A longer postprandial period for satiety evaluation after PGX consumption, greater than 120 min, may therefore result in higher satiety of the softgel compared to the granular form due to the combined effects of higher viscosity development from the softgel form slowing down digestion [36] and fermentation [39,41]. 

### 4.2. Postprandial Glycaemia

The lower peak blood glucose “spike” for PGX compared to WD may be in part due to the higher viscosity of PGX; slowing gastric emptying and reducing the rate of starch digestion and glucose absorption in the small intestine [36]. The higher blood glucose level for PGX at 120 min compared to the WD meal however, may have been due to the delayed release of glucose from starch digestion. The management and prevention of impaired glucose tolerance and type 2 diabetes requires the avoidance of postprandial “spikes” and a more gradual drop of blood glucose over time [42], thus the postprandial response for PGX indicates its potential benefits in relation to type 2 diabetes prevention and management. 

The glycaemia results from the present study are supported by those of Brand-Miller *et al.* [21,22] who reported that consumption of PGX with a bread meal was effective in reducing the postprandial glycaemic response and that consuming PGX with water, as part of a meal, reduced the GI of the meal. Additional support for the present findings on PGX can be found in studies of other viscous fibres, such as β-glucan, which improved postprandial glycaemia due to the fibre’s ability to increase the viscosity within the gut [43,44]. The ability of PGX to maintain plasma glucose concentrations after a standard meal within a relatively narrow range, compared to WD, suggests that this highly viscous soluble fibre may have a clinically significant role in long term blood glucose control which warrants further research, particularly in at-risk subjects. 

### 4.3. Trained Panel Results

The subjects in the present study were trained in an effort to carefully control factors other than the treatments which may influence satiety. Measurements relating to feelings of fullness are highly subjective and depend on a number of physiological, psychological, and social factors, which makes assessment of satiety challenging due to lack of precision. During training, all subjects consumed 45 g of breakfast cereal which resulted in a degree of fullness as recorded on the LMS and indicated the 45 g meal was sufficient to alleviate feelings of hunger in all of the subjects. In previous research by Solah *et al.* [30], training of the subjects improved the discriminatory power to detect differences in the satiety effect between PGX and inulin, and reduced between subject variability. Training may have contributed to the low standard error of mean (SEM) in this study (Figure 3). The low SEM for postprandial satiety for the PGX meal versus the WD meal is reflected in low SEM for postprandial glycaemia values. 

The LMS was considered to provide better discrimination of satiety sensations compared to a VAS for the trained panel used in this research. Furthermore, as a result of training, there is an expectation that the trained panel can detect differences in the feelings of fullness after consuming meals of different volumes or compositions. During training, “feelings of hunger” are considered separate to “feelings of fullness” so the “mean fullness score” was not adjusted using the fasting hunger score. The first reason for not adjusting the fullness score was related to the purpose of the training. For example, to adjust a fullness score of 3.5 for the WD meal to a minus value, using the fasting score, implies subjects were not feeling a degree of fullness at 15 min. Secondly, fasting triglycerides and satiety are more variable than postprandial triglycerides and satiety results [45,46,47]. Previous research has shown that fasting triglycerides levels have a high intra-individual day-to-day variability of 15% to 30% [45,46]. Although training was undertaken to carefully control factors other than the treatment which may influence satiety, the effect of difference related to gender and weight [48,49] may not be alleviated by training.

## 5. Conclusions

The trained satiety panel used in this research found that when PGX in both the granular and softgel form were consumed with a high carbohydrate breakfast, satiety was increased compared to WD. It was also determined that PGX in granular form provided significant reductions in the postprandial glucose response. This postprandial comparison of PGX and WD has provided additional knowledge on the connection between postprandial satiety and glycaemia responses due to fibre consumption and viscosity. The potential role of consumption of PGX in assisting the prevention of obesity and development of type 2 diabetes has been strengthened by the findings of this study. Research into the long-term effect of PGX on energy intake and blood glucose control, particularly in at risk groups, such as the obese or those with insulin resistance, requires further investigation. 

## Figures and Tables

**Table 1:** Nutrient composition of standard meal and water, excluding test fibre.

	Special K Original ^a^	Cornflakes ^b^	Full Cream Milk ^c^	Drinking Water	Total Meal
Mass of Serve (g)	22.5	22.5	175	500	720
Protein (g/serve)	4.4	1.7	5.6	0	117
Fat (g/serve)	0.2	0.6	6.3	0	7.1
Available Carbohydrates (g/serve)	15	18.1	8.3	0	41.4
Total Dietary Fibre (g/serve)	2.6	1.2	0	0	3.8
Energy (kJ)	350	347	508	0	1205

^a,b,c^ were combined prior to serving.

**Table 2:** Area under curve (AUC) for postprandial satiety and blood glucose levels.

**Satiety (Score (cm) Time (min))**		
	AUC mean (SD)	Difference from wheat dextrin Coefficient (95% CI), *p* value *
Wheat dextrin	215 (261)	
PGX softgel	477 (121)	262 (138, 387), *p* < 0.001
PGX (granules)	454 (242)	240 (183, 296), *p* < 0.001
**Glucose (mmol/L·min)**		
	AUC mean (SD)	Difference (PGX-wheat dextrin) Coefficient (95% CI), *p* value *
Wheat dextrin	713 (67)	
PGX (granules)	674 (56)	−40 (−59, −21), *p* < 0.001

* Results were derived from a mixed effect model.
